# Microwave Assisted Extraction of Bioactive Carbohydrates from Different Morphological Parts of Alfalfa (*Medicago sativa* L.)

**DOI:** 10.3390/foods10020346

**Published:** 2021-02-06

**Authors:** Daniela Alejandra Solarte, Ana Isabel Ruiz-Matute, Diana M. Chito-Trujillo, Maite Rada-Mendoza, María Luz Sanz

**Affiliations:** 1 Grupo de Investigación Biotecnología, Calidad Medioambiental y Seguridad Agroalimentaria (BICAMSA), Universidad del Cauca, Popayán 190003, Colombia; danielasolarte@unicauca.edu.co (D.A.S.); dchito@unicauca.edu.co (D.M.C.-T.); mrada@unicauca.edu.co (M.R.-M.); 2 Instituto de Química Orgánica General (IQOG-CSIC), Juan de la Cierva 3, 28006 Madrid, Spain; ana.ruiz@csic.es

**Keywords:** alfalfa (*Medicago sativa* L.), α-galactooligosaccharides (α-GOS), inositols, sugars, microwave assisted extraction (MAE), solid-liquid extraction (SLE)

## Abstract

Despite the nutritional properties of alfalfa, its production is mainly for animal feed and it is undervalued as a food source. In this study, the valorization of alfalfa as a potential source of bioactive carbohydrates [inositols, α-galactooligosaccharides (α-GOS)] is presented. A Box–Behnken experimental design was used to optimize the extraction of these carbohydrates from leaves, stems, and seeds of alfalfa by solid–liquid extraction (SLE) and microwave-assisted extraction (MAE). Optimal extraction temperatures were similar for both treatments (40 °C leaves, 80 °C seeds); however, SLE required longer times (32.5 and 60 min vs. 5 min). In general, under similar extraction conditions, MAE provided higher yields of inositols (up to twice) and α-GOS (up to 7 times); hence, MAE was selected for their extraction from 13 alfalfa samples. Pinitol was the most abundant inositol of leaves and stems (24.2–31.0 mg·g^−1^ and 15.5–22.5 mg·g^−1^, respectively) while seed extracts were rich in α-GOS, mainly in stachyose (48.8–84.7 mg·g^−1^). In addition, inositols and α-GOS concentrations of lyophilized MAE extracts were stable for up to 26 days at 50 °C. These findings demonstrate that alfalfa is a valuable source of bioactive carbohydrates and MAE a promising alternative technique to obtain functional extracts.

## 1. Introduction

Alfalfa or lucerne (*Medicago sativa*) is a perennial herbaceous plant belonging to the Fabaceae family. Worldwide, it is widely cultivated and intended for animal nutrition since it can be grown in areas with extreme abiotic factors [1] and has high protein and digestible fiber levels [2,3]. Alfalfa extracts have been proven to be an efficient dietary tool in the treatment of hypertension, metabolic disorders related to glucose, and lipid metabolism, arthritis, and kidney problems [2] and to be helpful in lowering cholesterol levels in both animals and humans [4,5]. These activities have been attributed to its content in several bioactive compounds such as sterols, triterpenes, phenolic compounds, fatty acids, and saponins [2,5,6,7,8]. While several studies have been carried out regarding these compounds, scarce attention has been paid to its bioactive carbohydrate composition [1,9].

Cyclitols (also named inositols) are minor phytochemicals with several reported biological activities (anti-diabetic, anti-inflammatory, antioxidant, and even anti-cancer properties) [1]. The presence of free inositols such as *myo*-inositol, methyl-inositols (such as pinitol and ononitol), and glycosyl-inositols (such as galactinol) have been previously detected in alfalfa leaves [9]. Alfalfa seeds contain several glycosyl-inositols and they have also been shown to be a rich source of α-galactooligosaccharides (α-GOS) from the raffinose family. α-GOS are considered prebiotics which selectively stimulate the growth and activity of a limited number of beneficial bacteria (mainly *Bifidobacterium* and *Lactobacillus*) in the colon [10,11,12]. Thus, alfalfa is a leguminous plant of great interest for exploitation by the food industry as a source of bioactive carbohydrates.

Extraction of bioactive carbohydrates from fruits, vegetables, and legumes have been usually carried out by conventional solid-liquid extraction (SLE) [13,14,15,16]. However, there is a growing interest in applying more efficient advanced extraction techniques which help to break vegetable tissues, releasing bioactive compounds from cell structures, as an alternative to the tedious and time-consuming SLE-based processes [17,18]. Among the most outstanding implemented techniques, Microwave Assisted Extraction (MAE) is preferred for its speed, efficiency, and safe operation [16,18,19,20,21]. During conventional treatments, heat energy is delivered through conduction–convection processes with the consequent loss of heat energy to the environment. However, during MAE, heating of the sample is produced in a targeted and selective process produced by the simultaneous combination of ionic conduction and dipole rotation which change microwave into thermal energy [22,23,24]. Subsequently, MAE provides shorter extraction times and more effective treatments due to the microwave properties—it can heat all samples simultaneously, without heating the vessel and with a faster energy transfer, reduce thermal gradients and unique heating selectivity, and ultimately afford better yields at lower costs [25,26,27,28].

The most relevant parameters that affect MAE treatments are solid–liquid ratio, extraction temperature, and time. Considering the high dependence among some of these parameters, MAE usually requires the optimization of the extraction conditions using experimental design approaches [29]. In closed systems, pressure is also an important variable, but it is directly dependent on temperature, it being preferable to control the latter to avoid degradation of thermolabile compounds [25]. Irradiation power can be also optimized; however, the number of extraction vessels simultaneously used mainly governs its selection. It is usually chosen as a compromise between minimizing the extraction time and avoiding solvent projections or degradation of thermolabile analytes [27]. The nature of the solvent also affects the extraction of bioactive carbohydrates; polar solvents such as water, methanol, or hydroalcoholic mixtures are commonly used. Based on their dielectric constant, water has a greater capacity to obstruct microwaves than other solvents such as methanol and, therefore, favors their penetration. On the contrary, methanol has a higher ability than water to dissipate the microwave energy as heat [30].

MAE has been mainly used for the extraction of polysaccharides (i.e., pectins, inulin, etc) from different natural matrices [20,31], although recently its utility for the extraction of bioactive low molecular weight carbohydrates (LMWC) from food residues has been addressed (i.e., lettuce leaves [18], artichoke bracts [20], and legume pods [21]). However, as far as we know, MAE efficiency for the extraction of bioactive carbohydrates from alfalfa has not yet been evaluated.

Therefore, in this work both SLE and MAE methods were optimized and compared for the effective extraction of cyclitols and α-GOS from different morphological parts of alfalfa. Thermal stability of lyophilized extracts obtained by the optimal extraction technique was also evaluated.

## 2. Material and Methods

### 2.1. Reagents

Analytical standards of pinitol, *myo*-inositol, galactinol, glucose, fructose, sucrose, raffinose, stachyose, and phenyl-*β*-D-glucopyranoside were purchased from Sigma Chemical Co. (St. Louis, MO, USA). Ethanol and methanol were acquired from Scharlab (Barcelona, Spain) and hydroxylamine chloride, hexamethyldisilazane and trifluoroacetic acid were purchased from Sigma Chemical Co. (St. Louis, MO, USA) 

### 2.2. Samples 

Thirteen samples from three different morphological parts: leaves (Lv1–Lv4), stems (St1–St4), and seeds (Sd1–Sd5)] of alfalfa (*Medicago sativa* L.) were purchased between June and August 2018. Leaves and stems were acquired in local markets of Nariño (Colombia), and seeds in Colombian and Spanish seed stores. The identification of samples is shown in Table S1 of Supplementary Material.

Leaves samples were dried on paper in the dark at room temperature for 72 h, while stems were cut into small pieces and dried at 40 °C in an oven for 24 h. Seeds were not subjected to any previous drying process. Samples were ground to fine particles using a domestic mill (Moulinex, Barcelona, Spain) and sieved through a 500 µm mesh. Finally, they were stored in a dry, hermetically sealed recipient protected from light until analysis at room temperature. 

### 2.3. Extraction Methods

Lv2 and Sd2 samples were selected for the optimization of both SLE and MAE methods. Prior to the evaluation of the effect of different extraction conditions on cyclitol and α-GOS yields, optimization of solvent was tackled by SLE. Samples (0.3 g) were mixed with 10 mL of different percentages of ethanol:water and methanol:water (0–100%; 25–75%; 50–50%; 75–25%; 100–0%, *v/v*) and stirred at 75 °C for 16 min. Assays were performed in triplicate.

MAE extractions were carried out on MARS6 equipment (CEM, Matthews, NC, USA) provided with optic fiber (MTS-300, CEM, Matthews, NC, USA) for temperature control. Microwave power was set at 900 W. Samples were accurately weighed and placed along with a fixed volume of 10 mL of the selected solvent in 100 mL X-Press 1500 vessels (CEM, Matthews, NC, USA) and subjected to MAE (*n* = 3).

A Box–Behnken experimental design was performed in order to evaluate the effect of three independent variables (sample amount (*s*, g); temperature (*T*, °C) and time (*t*, min)) on extraction of cyclitols and α-GOS by SLE and MAE. These independent variables were selected as the most relevant factors according to findings in previous investigations [16,20,21]. Sample amount was varied to evaluate the effect of modifying the solid liquid ratio, using a fixed solvent volume of 10 mL (the minimum allowed by the X-Press 1500 vessels). A total of 15 experiments were carried out in random order and three central points were included to estimate the experimental error (Table 1). 

Experimental ranges for factors evaluated were: *T* = 40, 80, 120 °C, *t* = 5, 32.5, 60 min, and *s* = 0.1, 0.3, 0.5 g. Each experiment was carried out in triplicate. Response surface methodology (RSM) was used to calculate the coefficients of *R*_1_, the total concentration of extracted cyclitols (mg·g^−1^) and *R*_2_, the total concentration of α-GOS (mg·g^−1^), both responses to be maximized, in the model proposed for leaves and seeds and to estimate the statistical significance of the regression coefficients. The quadratic model proposed was:

*R* = β_0_ +β_1_*T* +β_2_*t* +β_3_*s* +β_1,1_*T*^2^ +β_2,2_*t^2^* +β_3,3_*s*^2^ +β_1,2_*Tt* +β_1,3_*Ts* +β_2,3_*ts* + ε (1)

where β_0_ is the intercept, β*_i_* are the first-order coefficients, β*_i,i_* the quadratic coefficients for *i*th factors, β*_i,j_* the coefficients for interaction between the factors *i* and *j* and ε is the error. *R*_1_ and *R*_2_ were estimated by multiple linear regression (MLR) using StatGraphics Centurion XV software (Statistical Graphics Corporation, Rockville, MD, USA). A multiple response analysis, that simultaneously maximized *R*_1_ and *R*_2,_ was also considered in seeds for selection of optimal SLE and MAE operating conditions; this function takes values between 0 (completely undesirable value) and 1 (completely desirable or ideal response).

After extraction procedure, samples were cooled in ice for 5 min and solid residue was removed by centrifugation at 4400× *g* at 10 °C for 10 min. A clear solution was obtained and kept at −18 °C until analysis by gas chromatography-mass spectrometry (GC‑MS). Each procedure was performed in triplicate.

### 2.4. GC-MS Analysis

Prior to the GC-MS analysis of inositols and α-GOS the alfalfa extracts were derivatized to form their corresponding trimethylsilyl oximes (TMSO) according to Ruiz-Aceituno et al. [20]. Extracts (0.5 mL) and 0.1 mL of internal standard (phenyl-*β*-D-glucopyranoside, 1 mg·mL^−1^) were dried under vacuum in a miVac concentrator (Inycom, Madrid, Spain) at 40 °C. Oximes were prepared by addition of 350 µL of a 2.5% hydroxylamine chloride in pyridine solution heating at 75 °C for 30 min. Then, trimethylsilyl derivatives were obtained by addition of 350 µL of hexamethyldisilazane and 35 µL of trifluoroacetic acid at 45 °C for 30 min. Samples were centrifuged at 4400× *g* for 10 min and the supernatant was recovered. 

GC-MS analysis was carried out using a 6890 N gas chromatograph coupled to a 5973 N quadruple mass detector (Agilent Technologies, Santa Clara, CA, USA) with a high-temperature capillary column HT-5 coated with 5% phenyl polycarborane-siloxane (25 m × 0.22 mm id, 0.10 μm film thickness; Analytical Science, Santa Clara, CA, USA) and using helium at 0.8 mL·min^−1^ as carrier gas. The oven temperature was programmed as follows: 160 °C (10 min), then at 10 °C·min^−1^ to 380 °C, which was maintained for 5 min. Injections (1 μL) were carried out in split mode (1:20) at 280 °C. Mass spectrometer transfer line and ion source were set at 280 °C and 230 °C, respectively. Mass spectra were acquired in electron impact mode at 70 eV, scanning in a mass range: 40–700 *m/z*. Data acquisition was conducted using HP ChemStation software (Agilent Technologies, Santa Clara, CA, USA).

Identification of cyclitols and α-GOS was carried out by comparison of their corresponding linear retention indices (*I^T^*) and retention times and mass spectra with those of corresponding standards and data reported in the literature [13]. When analytical standards were not available, compounds were tentatively identified based on their chromatographic retention and mass spectral data. Quantitative data were determined by the internal standard method. Standard solutions of target compounds over the expected concentration range in sample extracts (sugars and inositols from 0.005 to 0.5 mg·mL^−1^, and stachyose from 0.0025 to 1.0 mg·mL^−1^) were used to calculate the response factors relative to the internal standard (*n* = 3). Ononitol, trehalose, digalactosyl-inositol, and galactose were quantified from external calibration curve of pinitol, sucrose, galactinol, and glucose, respectively.

### 2.5. Thermal Stability 

Aliquots of 1 mL of Lv2 and Sd2 extracts obtained by MAE were lyophilized in a Cryodos −80 freeze dryer (Telstar S.A., Madrid, Spain) at −80 °C and 0.080 mbar and then, subjected to accelerated temperature conditions at 50 °C in a convection oven for 26 days, following the process described by Rodríguez-Sánchez et al. [32]. Samples from each extract were collected at 0, 5, 12, 19, and 26 days, reconstituted with Milli-Q water (1 mL) and analyzed by GC-MS, as described in previous section. 

### 2.6. Statistical Analysis

The analysis of variance ANOVA was used to determine significant differences (*p* ≤ 0.05) between different samples, using the StatGraphics Centurion XV software (Statistical Graphics Corporation, Rockville, MD, USA).

## 3. Results and Discussion 

### 3.1. Bioactive Carbohydrates Composition of Alfalfa Leaves, Stems and Seeds

**Figure 1:**
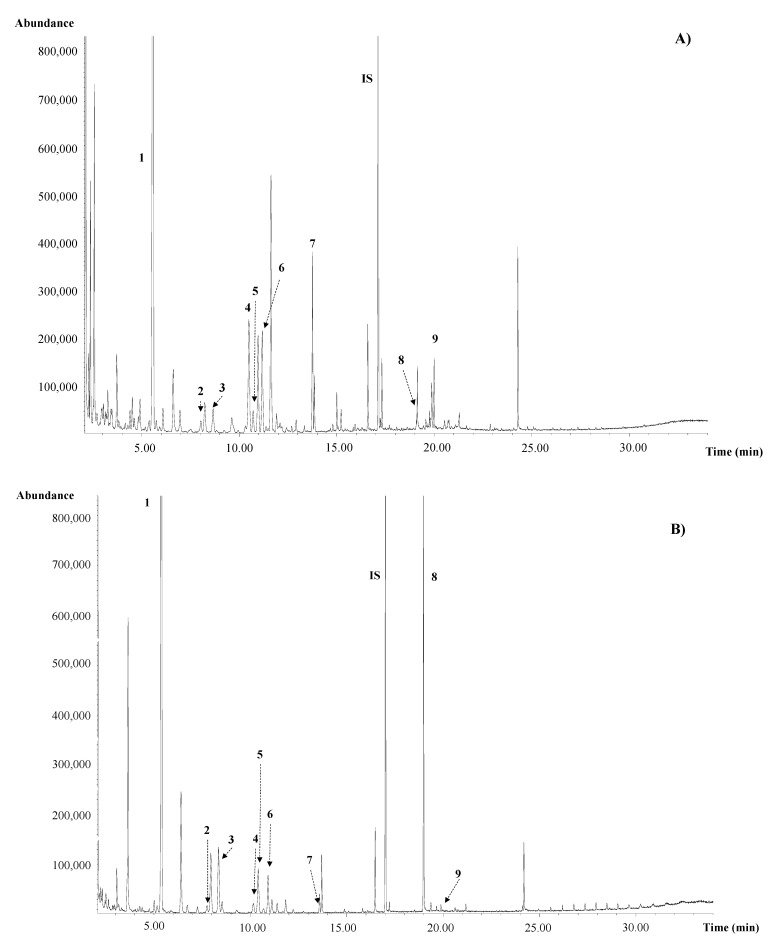

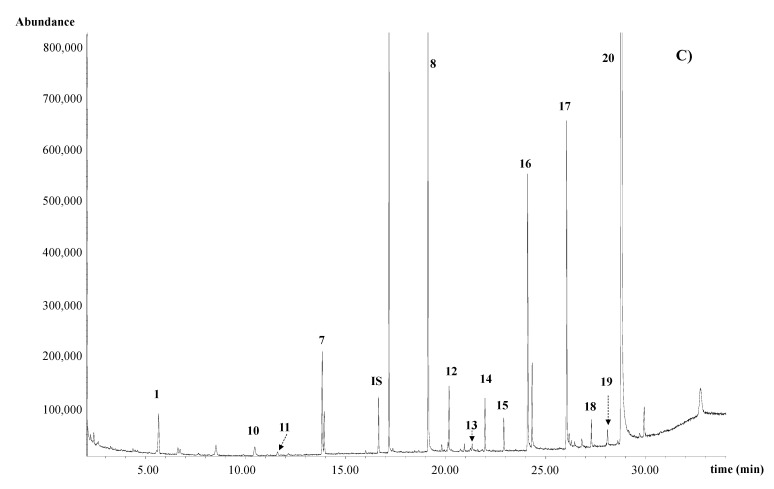
GC-MS profile of alfalfa SLE extracts previously derivatized to TMS-Oximes. (**A**) Leaves (Lv2); (**B**) Stems (St2); (**C**) Seeds (Sd2): 1: pinitol; 2: fructose 1; 3: fructose 2; 4: ononitol 5: glucose *E*; 6: glucose *Z*; 7: *myo*-inositol; 8: sucrose; 9: trehalose; 10: galactose *E*; 11: galactose *Z*; 12: galactosyl-pinitol A; 13: galactosyl-pinitol B; 14: galactinol; 15: digalactosyl-glycerol, 16: raffinose; 17–19: digalactosyl-inositol; 20: stachyose; IS: internal standard.

Chromatographic profiles of Lv2, St2, and Sd2 extracts obtained by SLE at 75 °C for 16 min using 0.3 g of sample and 10 mL of Milli-Q water are shown in Figure 1; carbohydrate composition of Lv2, St2, and Sd2 extracts, indicating their *t_R_* and experimental *I^T^* is detailed in Table S2 of Supplementary Material. In general, similar GC profiles were observed for leaves and stems extracts (Figure 1A,B), while those of seeds (Figure 1C) showed a different carbohydrate composition. Several free inositols such as pinitol, ononitol and *myo*-inositol were detected in leaves and stems, while seeds were a richer source of glycosyl-cyclitols such as galactosyl-pinitols, galactinol, and digalactosyl-cyclitols, as previously reported by Horbowicz et al. [9]. Other LMWC such as fructose, glucose, galactose, sucrose, and trehalose were also present in leaves and stems. Sucrose was also found in seeds extracts together with the α-GOS raffinose and stachyose. Galactinol has been described to be the main galactosyl donor implicated in α-GOS biosynthetic pathway, where stachyose is synthesized from raffinose, and this trisaccharide from sucrose [33]. These oligosaccharides, which were not detected in leaves and stems, have been related to maturation, desiccation tolerance, and storability of vegetable seeds, reducing damage caused by oxidative stress or ageing [9,33]. Considering the similar composition of leaf and stem extracts, only Lv2 and Sd2 samples were chosen for the subsequent analyses.

### 3.2. Optimization of SLE Method

**Figure 2:**
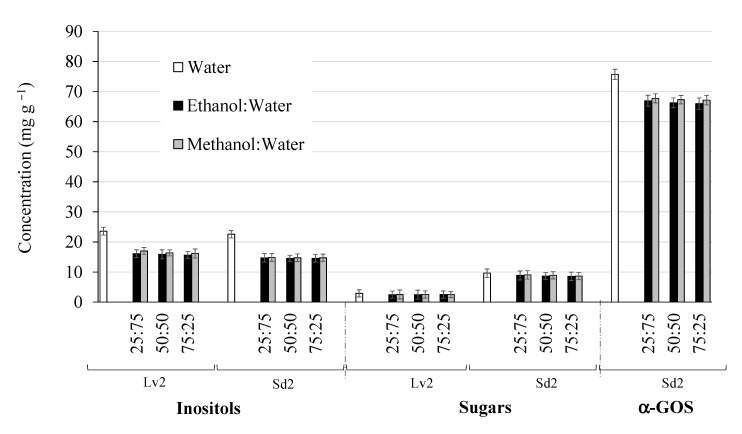
Inositol, sugar and α-GOS yields of Lv2 and Sd2 SLE extracts obtained using different extraction solvents. Lv2, leaves; Sd2, seeds; α-GOS, α-galactooligosaccharides

Firstly, different solvents for the extraction of bioactive carbohydrates from alfalfa leaves (Lv2) and seeds (Sd2) by SLE were evaluated. This is a crucial step for the optimization of an extraction method since it has environmental, social, and economic implications. The use of GRAS (generally recognized as safe) solvents instead of contaminant and hazardous solvents is encouraged. Moreover, solvent price and recyclability are other parameters to be considered [34]. Polar solvents are the most appropriate for the extraction of carbohydrates, considering their high polarity. Moreover, these solvents have further application in MAE since they strongly absorb microwave energy due to the presence of permanent dipoles [17]. Then, water and different percentages of ethanol:water and methanol:water were assayed within an extraction cycle of 16 min at 75 °C under stirring conditions. Figure 2 depicts the carbohydrate yields extracted from leaves and seeds using these solvents. To simplify data, carbohydrates have been grouped in inositols, α-GOS and non-bioactive sugars (glucose, galactose, fructose and sucrose). Regarding inositols, no noticeable differences were observed among the different percentages of methanol:water and ethanol:water assayed for both leaves and seeds. However, yields obtained with aqueous extracts (100% water) were higher than those of alcoholic mixtures. Moreover, water extracts also presented the greatest yields of α-GOS in alfalfa seeds (up to 11% higher than yields achieved using alcohol:water mixtures). These results are in good agreement with those reported by López-Molina et al. [35] and Ruiz-Aceituno et al. [14], for the SLE of prebiotics from artichoke and inositols from pine nuts, respectively. Furthermore, from a technical point of view, the choice of water as the extraction solvent is considered the most appropriate due to its availability at low cost, its versatility in different environmental conditions and its properties as a green solvent [34,36]. On the contrary, in a study of Carrero-Carralero et al. [16], although water was the most effective solvent for the extraction of inositols and prebiotics from mung beans, it was not selected due to the high concentrations of non-bioactive sugars that were also co-extracted. This was not the case for alfalfa extractions, in which similar extraction yields of non-bioactive sugars were obtained using the different evaluated solvents for both leaves and seeds. Considering the relative low concentrations of sugars in alfalfa compared with those of bioactive carbohydrates, water was finally selected as a solvent for further studies.

After selection of the solvent, a complete optimization of SLE was carried out. A total of 15 experiments were done in randomized order according to a Box–Behnken design to study the impact of the three independent variables (extraction time (*t*), temperature (*T*) and sample amount (*s*) for a fixed solvent volume of 10 mL) on the efficiency of the SLE of total inositol (*R*_1_, mg·g^−1^) concentrations in alfalfa leaves and of both total inositol and total α-GOS (*R*_2_, mg·g^−1^) in alfalfa seeds. Sugar concentrations were also considered in order to control a potential and undesirable substantial increase in their concentration under certain extraction conditions. Table 1 shows the obtained yields at the different evaluated conditions. While a variation of 2.7 times was found for inositol and sugar concentrations of Sd2 depending on the extraction conditions (inositols between 11.5 and 31.2 mg·g^−1^ and sugars between 6.6 and 18.2 mg·g^−1^), concentrations of α-GOS underwent a variation of almost 16 times (between 11.4–190.8 mg·g^−1^). Regarding leaves extract, a slight variation was observed for inositol concentrations (most extracts presented values between 14.3 and 21.8 mg g^−1^, except for experiment 15 which showed 31.4 mg g^−1^), while sugar concentrations varied from 2.98 to 9.0 mg·g^−1^. In general, at higher temperatures and extraction times, higher inositol and α-GOS concentrations were obtained. 

Response surface methodology was followed to calculate the coefficients of *R*_1_ and *R*_2_ in the proposed quadratic model and to estimate the statistical significance of the regression coefficients. Regarding inositols (*R*_1_), *t^2^* was the most significant factor at a 95% confidence level (*p* < 0.05) for Lv2 extract, while *s*, *s*^2^ and *t* were the factors statistically significant (*p* < 0.05) for Sd2 extract. Regarding α-GOS (*R*_2_) for Sd2 extract, *s* and *t* were the most significant factors. The quadratic regression equations (from Equation (1)) for *R*_1_ and *R*_2_ are shown in Table 2. The surface plots to maximize *R*_1_ in leaves and *R*_1_ and *R*_2_ in seeds after excluding no significant (*p* > 0.05) terms in the model are shown in Figure S1A–C of Supplementary Material. Good fits were found for both inositols and α-GOS in Sd2 extract, where the *R*^2^ were 0.97 and 0.67, respectively. The resultant optimal conditions which maximized inositols and α-GOS yields were 80 °C, 60 min, 0.1 g, and 80 °C, 60 min, 0.5 g, respectively. On the contrary, a low fit quality of the proposed model was found for inositols in Lv2 (*R*^2^ = 0.58), probably due to the low variability in their concentrations among experiments. Then, SLE conditions for extraction of inositols from Lv2 were selected considering the experiment that provided the highest inositol yields from those evaluated (Table 1, experiment 15: 40 °C, 32.5 min and 0.5 g).

Finally, a multiple response analysis that simultaneously maximized the extraction of inositols and α-GOS (*R*_1_ and *R*_2_) in Sd2 extract was carried out. Optimal conditions were 80 °C, 60 min, and 0.5 g. 

### 3.3. Optimization of MAE Method

Water was selected as the extraction solvent for MAE experiments, considering the results obtained during the SLE treatment of alfalfa leaves and seeds, its high capacity to obstruct microwaves which favor their penetration [30], and its green nature. Similar to SLE procedure, optimization of MAE conditions (*T*, *t* and *s*) was carried out following a Box–Behnken design. Table 1 shows the experimental results of inositol and α-GOS concentrations obtained for the 15 assays performed. A slight variation of inositol concentrations depending on the extraction conditions applied was also found for Lv2 sample (between 26.2 and 38.4 mg·g^−1^), while changes in inositol and α-GOS concentrations of Sd2 were greater (between 13.5 and 30.9 mg·g^−1^ and between 40.6 and 270.6 mg·g^−1^, respectively). In general, under similar time and temperature conditions, inositol extraction yields in Sd2 were higher in those experiments carried out using lower sample amounts (e.g., experiment 9: 29.5 mg·g^−1^ vs. experiment 15: 13.5 mg·g^−1^). On the contrary, similar extraction yields were obtained by varying the time (for the same temperature and quantity of sample), probably due to the fact that 5 min was enough to achieve the maximum concentration of inositols under these conditions. Regarding α-GOS, higher concentrations were obtained when temperature increased. As previously reported by Alexandru et al. [28], at higher temperatures, solvent viscosity decreased which enhances the diffusivity and then the extraction efficiency. Regarding sugars, they varied from 3.25 to 8.36 mg·g^−1^ in Lv2 and from 9.78 to 17.39 mg·g^−1^ in Sd2.

However, it is difficult to evaluate the influence of extraction parameters independently; thus, response surface methodology was used to calculate the coefficients of *R*_1_ and *R*_2_ and to estimate the statistical significance of the regression coefficients. In this case, *s* and *Tt* were the most statistically significant factors (*p* < 0.05) for maximization of *R*_1_ in Lv2 sample, while *s* and *s*^2^ were those for maximization of *R*_1_, and *s* and *ts* for *R*_2_ in Sd2 extract. The quadratic equations, after excluding non-significant parameters (*p* > 0.05), can be observed in Table 2 (response surface plots are shown in Figure S1D–F of Supplementary Material). According to SLE results, low fit quality of the quadratic model was found for inositols in Lv2 extract (*R*^2^ = 59%), probably considering the low variability of the total inositols detected under different experimental conditions. Considering the low efficiency of the model, the experiment 2 (40 °C, 5 min, and 0.3 g) was selected as MAE optimal conditions taking into account that it provided high inositol yields (Table 1), minimizing the temperature and extraction time, and therefore the energy consumption. 

The quadratic model for Sd2 sample appropriately described the variability of *R*_1_ and *R*_2_ (*R*^2^ = 99% and 66%, respectively). Then, the optimal conditions for maximization of inositols were: 80 °C, 5 min, 0.1 g, and for α-GOS were: 80 °C, 60 min, 0.5 g. The multiple response analysis performed to maximize the extraction of inositols and α-GOS (*R*_1_ and *R*_2_) by MAE displayed as optimal conditions: 80 °C, 5 min, and 0.5 g. Thus, these conditions were selected for further experiments with alfalfa seeds.

### 3.4. Comparison of SLE vs. MAE

The efficiency of extraction method could be established by comparison of bioactive inositols, α-GOS, and other sugar yields obtained at similar experimental conditions assayed (Table 1). Inositol concentrations obtained by MAE were, in general, higher than those obtained by SLE in both Lv2 and Sd2, A similar behavior was also observed for α-GOS concentration in seeds. Regarding sugar content, non-significant differences were found for leaves samples by both techniques, contrarily to that observed for seeds. Nevertheless, these concentrations were lower than those of inositols and α-GOS in most cases. Then, the implementation of an additional fractionation step for the subsequent removal of sugars was not considered mandatory. 

Regarding the extraction of bioactive carbohydrates under optimal conditions, MAE required shorter extraction times for both leaves and seeds (MAE 5 min vs. SLE 32.5 min Lv2 and 60 min Sd2) and smaller amounts of samples for leaves (MAE 0.3 g vs. SLE 0.5 g) than SLE. Similar temperatures were selected as optimal for both treatments (80 °C for seeds and 40 °C for leaves). Under these optimal conditions, significant higher inositol yields in both leaves and seeds were obtained by MAE (see concentration values marked in bold in Table 1), however, greater concentrations of α-GOS were obtained by SLE.

From these results, although both techniques could be appropriate to extract bioactive carbohydrates from alfalfa, considering the advantages of MAE in terms of extraction times vs. inositols and α-GOS yields, this technique was selected for further experiments. These results were in good agreement with those reported by Carrero-Carralero et al. [16], who found that MAE provided similar bioactive carbohydrates yields than SLE, but with shorter extraction times. Similar results were also observed by Zuluaga et al. [21] for the extraction of inositols from legume pods. 

### 3.5. Analysis of Bioactive Carbohydrates of Alfalfa Samples

Optimal MAE conditions were applied to obtain extracts enriched in inositols and α-GOS from leaves (Lv1, Lv2, Lv3, and Lv4) and seeds samples (Sd1, Sd2, Sd3, Sd4, and Sd5). Extraction conditions of leaves were also used for stems (St1, St2, St3 and St4) considering their similar composition. Table 3 shows the concentrations of bioactive carbohydrates and sugars present in the analyzed samples. Leaves and stems were mainly constituted by inositols (total concentrations between 27.0 and 37.0 mg·g^−1^ for leaves and 17.9 and 24.9 mg·g^−1^ for stems). Pinitol was the most abundant inositol (24.2–31.0 mg·g^−1^ in leaves and 15.5–22.5 mg·g^−1^ in stems), mainly in Lv1 and Lv2. In general, similar concentrations of ononitol and *myo*-inositol were detected in all leaves and stems samples. Pinitol, ononitol, and *myo*-inositol concentrations were similar to that reported by Horbowicz et al. [9] and higher than those reported by Al-Suod et al. [15] for alfalfa leaves and stems after soxhlet ethanolic extraction. Moreover, these last authors did not detect ononitol in these samples, although they reported the presence of D-*chiro* and *scyllo*-inositols, non-identified in the present work. Regarding sugars, low concentrations were found for glucose, fructose, and sucrose in all leaves and stems samples. While similar concentrations of glucose and fructose were extracted by Horbowicz et al. [9] from old leaves using ethanol:water (1:1, *v/v*) at 80 °C, higher sucrose contents were found by these authors. 

Seed extracts were rich in α-GOS (56.7–93.1 mg·g^−1^), stachyose was the most abundant (48.8–84.7 mg·g^−1^), mainly in Sd3 extract. Significant differences were observed in stachyose concentrations among the different seed samples. However, these values (mainly in Sd1 and Sd4) were similar to those reported by Horbowicz et al. [9]; samples Sd2, Sd3, and Sd5 presented higher concentrations. Pinitol (2.3–2.7 mg·g^−1^) and galactinol (5.1–5.5 mg·g^−1^) contents were also higher than those reported by Horbowicz et al. [9]. These higher yields of bioactive carbohydrates found in this work could be due to the higher efficiency of the developed MAE method in comparison with the SLE method used by Horbowicz et al. [9].

### 3.6. Stability Study

In order to evaluate the thermal stability of obtained extracts due to their potential use as food ingredient, Lv2 and Sd2 extracts were lyophilized and stored at 50 °C for 26 days. The variability of inositols and α-GOS concentrations over time is shown in Table 4. Contents of inositols in both Lv2 and Sd2 extracts showed a slight decrease during storage time (from 36.8 to 34.5 mg·g^−1^ and from 28.3 to 27.2 mg·g^−1^, respectively). Similarly, α-GOS concentrations decreased less than 5% after 26 days (from 83.2 mg·g^−1^ to 79.1 mg·g^−1^). On the contrary a marked decrease was observed in sugar concentrations of these samples (between 19.7% and 24.4%); this significant decrease could be due to the degradation of these carbohydrates to give rise to organic acids, hydroxymethylfurfural, and unwanted colored compounds, among others [37,38]. A similar behavior (stability of inositols and decrease of sugars) has been previously observed during storage of other matrices (e.g., dried fruits, [39]). Stability of free inositol has been attributed to the absence of non-reducing groups in their molecule, which seems to confer stability during thermal treatments [40]. Alterations experimented by bioactive carbohydrates during storage were not drastic, so these extracts had an acceptable stability to be used as food ingredients.

## 4. Conclusions

Optimized SLE and MAE methods to obtain extracts rich in inositols and α-GOS from different morphological parts of alfalfa (leaves, stems, and seeds) are proposed. Although both techniques were appropriate to extract bioactive carbohydrates from alfalfa using water as GRAS solvent, MAE presented advantages in terms of extraction times vs. inositols and α-GOS yields. Moreover, in the case of leaves, MAE also provided similar inositol concentrations to the classical treatment from less raw material. The increased speed and the efficiency of the extraction process by microwaves is advantageous considering the lower consumption of energy and, thus, the costs, which should be taken into account for the scaling up of the extraction process. Thus, the MAE methodology here proposed could be considered a promising efficient and green strategy to obtain bioactive carbohydrate extracts to be used as functional food ingredients. Moreover, this work opens new routes of revalorization of alfalfa (leaves, stems, and seeds) as a potential source of bioactive carbohydrates, such as inositols and α-GOS, of interest for the agri- food industry.

## Figures and Tables

**Table 1:** Experimental Box–Behnken design and inositols, sugars, and α-GOS content obtained by SLE and MAE (mg·g^−1^). Standard deviation in brackets (*n* = 3).

No	*t* (min)	*T* (°C)	*s* (g)	Inositols (mg·g^−1^)				Sugars (mg·g^−1^)				α-GOS (mg·g^−1^)	
				Leaves		Seeds		Leaves		Seeds		Seeds	
				SLE	MAE	SLE	MAE	SLE	MAE	SLE	MAE	SLE	MAE
1	32.5	80.0	0.3	21.5 (1.0) ^b^	29.6 (1.3) ^a^	16.9 (0.6) ^a^	14.7 (0.4) ^b^	3.5 (0.1) ^b^	4.8 (0.1) ^a^	14.1 (0.1) ^a^	12.7 (0.4) ^b^	84.7 (0.7) ^b^	107.0 (2.1) ^a^
2	5.0	40.0	0.3	18.4 (0.8) ^b^	36.2 (0.6) ^a,#,*^	11.5 (0.4) ^b^	14.6 (0.5) ^a^	3.2 (0.1) ^a^	**3.8 (0.1) ^a,#^**	6.67 (0.2) ^b^	12.4 (0,2) ^a^	11.4 (0.4) ^b^	78.6 (1.9) ^a^
3	5.0	80.0	0.5	14.7 (0.6) ^b^	30.9 (1.3) ^a^	12.8 (0.3) ^b^	**13.7** **(0.4) ^a,#^**	3.4 (0.1) ^a^	3.3 (0.1) ^a^	12.5 (0.5) ^b^	**15.1 (0.4) ^a,#^**	113.7 (3.5) ^b^	**138.4** **(2.6) ^a,&^**
4	60.0	120.0	0.3	14.6 (0.4) ^b^	30.8 (0.9) ^a^	17.9 (0.4) ^a^	16.8 (0.8) ^a^	5.1 (0.1) ^a^	4.8 (0.1) ^a^	14.9 (0.3) ^a^	13.3 (0.6) ^b^	155.2 (6.0) ^a^	91.4 (0.8) ^b^
5	32.5	120.0	0.1	15.5 (0.1) ^b^	37.5 (1.8) ^a^	28.4 (0.4) ^b^	29.5 (0.6) ^a^	6.82 (0.02) ^a^	6.7 (0.1) ^a^	8.859 (0.4) ^b^	9.8 (0.4) ^a^	50.6 (2.0) ^b^	62.4 (0.4) ^a^
6	32.5	80.0	0.3	20.7 (1.1) ^b^	31.7 (1.3) ^a^	14.6 (0.4) ^a^	14.7 (0.5) ^a^	5.2 (0.1) ^a^	4.9 (0.1) ^a^	13.7 (0.2) ^a^	11.6 (0.3) ^b^	80.3 (1.2) ^b^	91.1 (0.5) ^a^
7	5.0	80.0	0.1	20.1 (0.3) ^b^	30.9 (1.2) ^a^	27.5 (0.4) ^b^	29.4 (0.5) ^a^	9.02 (0.02) ^a^	8.4 (0.1) ^b^	9.1 (0.3) ^a^	9.8 (0.6) ^a^	28.1 (0.7) ^b^	64.0 (0.7) ^a^
8	32.5	120.0	0.5	21.8 (0.7) ^b^	29.7 (1.3) ^a^	11.6 (0.4) ^b^	14.0 (0.5) ^a^	3.69 (0.04) ^a^	3.4 (0.1) ^a^	11.5 (0.5) ^b^	16.7 (0.8) ^a^	84.4 (0.6) ^b^	119.5 (1.0) ^a^
9	32.5	40.0	0.1	20.4 (0.4) ^b^	38.4 (0.6) ^a^	28.2 (0.5) ^b^	29.5 (0.9) ^a^	5.8 (0.1) ^b^	7.0 (0.2) ^a^	10.8 (0.4) ^b^	12.1 (0.2) ^a^	36.3 (0.5) ^b^	40.6 (0.2) ^a^
10	5.0	120.0	0.3	19.5 (0.3) ^b^	29.1 (1.2) ^a^	14.8 (0.3) ^b^	17.5 (0.03) ^a^	5.2 (0.1) ^a^	5.0 (0.1) ^a^	12.3 (0.3) ^b^	15.4 (0.1) ^a^	99.0 (1.1) ^b^	154.1 (0.6) ^a^
11	60.0	80.0	0.1	14.3 (0.5)^b^	29.8 (0.2) ^a^	31.2 (0.8) ^a^	30.9 (0.4) ^a^	6.79 (0.02) ^a^	6.05 (0.01) ^a^	16.7 (0.4) ^a^	11.7 (0.2) ^b^	96.1 (2.9) ^a^	97.0 (0.8) ^a^
12	32.5	80.0	0.3	21.1(0.02) ^b^	29.3 (1.1) ^a^	15.8 (0.3) ^a^	13.9 (0.1) ^a^	5.34 (0.03) ^a^	5.1 (0.1) ^a^	13.9 (0.4) ^a^	12.5 (0.5) ^b^	81.1 (1.6) ^b^	106.2 (0.9) ^a^
13	60.0	40.0	0.3	14.3 (0.4) ^b^	26.2 (0.2) ^a^	15.7 (0.4) ^a^	15.7 (0.9) ^a^	3.0 (0.1) ^b^	5.2 (0.2) ^a^	18.2 (0.5) ^b^	13.5 (0.2) ^a^	76.3 (0.4) ^b^	101.0 (2.3) ^a^
14	60.0	80.0	0.5	16.8 (0.8) ^b^	27.3 (1.0) ^a^	**12.5 (0.2) ^b,&^**	13.6 (0.3) ^a^	3.72 (0.04) ^a^	3.25 (0.04) ^a^	**13.5 (0.5) ^b,&^**	14.6 (0.6) ^a^	**190.8 (2.1) ^b,#^**	270.6 (2.4) ^a^
15	32.5	40.0	0.5	**31.4 (0.4) ^a,&^**	28.5 (1.1) ^b^	12.2 (0.4) ^b^	13.5 (0.2) ^a^	**4.4 (0.1)** **^a,#^**	4.1 (0.1) ^a^	11.8 (0.5) ^b^	17.4 (0.6) ^a^	156.6 (0.8) ^b^	186.0 (1.7) ^a^

^a,b^ Different letters indicate significant differences (*p* < 0.05) for the same experiment, analyte (inositols, sugars, and α-GOS) and sample (Lv2 and Sd2), **^#,&^** Different symbols indicate significant differences (*p* < 0.05) for the same analyte (inositols, sugars, and α-GOS) and sample (Lv2 and Sd2) under optimal extraction conditions for each technique. ^*^ Optimal conditions for each treatment are marked in bold. *t*, time; *T,* temperature; *s*, sample amount; SLE, solid-liquid extraction; MAE, Microwave Assisted Extraction; α-GOS, α-galactooligosaccharides

**Table 2:** Regression equations to maximize the extraction of inositols (*R*_1_) and α-GOS (*R*_2_) from leaves (Lv2) and seeds (Sd2) by solid-liquid extraction (SLE) and microwave assisted extraction (MAE).

		SLE		MAE	
	Response Variable	Model Equation	*R* ^2^	Model Equation	*R* ^2^
*Leaves*	*R*_1_	*R*_1_ = 4.174 + 0.066*T + 0.566*t + 52.000*s − 0.459*Ts − 0.087*t^2^	57.8	*R*_1_ = 43.614 − 0.086*T − 0.270*t − 12.625*s + 0.003*Tt	59.2
*Seeds*	*R*_1_	*R*_1_ = 38.005 + 0.049*t − 120.411*s + 131.518*s^2^	97.2	*R*_1_ = 42.741 − 0.082*t − 136.683*s+ 0.001*t^2^ + 160.721*s^2^	98.8
	*R*_2_	*R*_2_ = −12.385 + 1.210*t + 209.000*s	66.4	*R*_2_ = 54.903 − 0.785*t + 135.017*s + 4.509*ts	65.7

**Table 3:** Inositol, α-GOS and other low molecular weight carbohydrate content (mg·g^−1^) of MAE leaves, stems and seeds alfalfa extracts. Standard deviation in brackets (*n* = 3).

	Leaves				Stems				Seeds				
	Lv1	Lv2	Lv3	Lv4	St1	St2	St3	St4	Sd1	Sd2	Sd3	Sd4	Sd5
**Pinitol**	31.0 ^a^ (1.4)	30.1 ^a^ (0.4)	24.3 ^b^ (0.6)	24.2 ^b^ (0.1)	19.3 ^b^ (1.2)	22.5 ^a^ (0.9)	15.5 ^c^ (0.8)	19.5 ^b^ (0.6)	2.3 ^c^ (0.1)	2.5 ^b^ (0.1)	2.7 ^a^ (0.1)	2.3 ^c^ (0.1)	2.5 ^b^ (0.1)
**Fructose**	0.7 ^d^ (0.1)	0.9 ^c^ (0.1)	1.7 ^a^ (0.1)	1.5 ^b^ (0.1)	1.1 ^d^ (0.1)	3.0 ^a^ (0.2)	1.4 ^c^ (0.1)	1.8 ^b^ (0.2)	-	-	-	-	-
**Ononitol**	1.0 ^c^ (0.1)	4.6 ^a^ (0.1)	1.6 ^b^ (0.1)	1.1 ^c^ (0.1)	1.1 ^b^ (0.1)	1.4 ^a^ (0.1)	1.4 ^a^ (0.1)	1.0 ^b^ (0.1)	-	-	-	-	-
**Glucose**	0.9 ^d^ (0.1)	2.4 ^a^ (0.1)	1.2 ^b^ (0.1)	1.0 ^c^ (0.1)	1.2 ^c^ (0.1)	2.4 ^a^ (0.1)	1.5 ^b^ (0.1)	1.1 ^c^ (0.1)	-	-	-	-	-
***myo*-Inositol**	1.1 ^c^ (0.1)	2.3 ^a^ (0.1)	1.6 ^b^ (0.1)	1.7 ^b^ (0.1)	1.0 ^a^ (0.1)	1.0 ^a^ (0.1)	1.0 ^a^ (0.1)	1.1 ^a^ (0.1)	2.7 ^b^ (0.1)	3.1 ^a^ (0.1)	3.2 ^a^ (0.1)	2.8 ^b^ (0.1)	3.1 ^a^ (0.1)
**Sucrose**	-	-	1.9 ^a^ (0.1)	1.7 ^b^ (0.1)	2.8 ^a^ (0.1)	2.6 ^b^ (0.1)	1.7 ^d^ (0.1)	2.4 ^c^ (0.1)	11.7 ^b^ (0.1)	10.5 ^d^ (0.1)	11.0 ^c^ (0.4)	12.5 ^a^ (0.1)	12.5 ^a^ (0.2)
**Galactinol**	-	-	-	-	-	-	-	-	5.2 ^c,d^ (0.1)	5.3 ^b,c^ (0.3)	5.5 ^a^ (0.1)	5.1 ^d^ (0.1)	5.4 ^a,b^ (0.1)
**Digalactosyl-inositol**	-	-	-	-	-	-	-	-	5.1 ^c^ (0.1)	6.4 ^a^ (0.4)	5.3 ^b,c^ (0.1)	5.2 ^c^ (0.1)	5.4 ^b^ (0.2)
**Raffinose**	-	-	-	-	-	-	-	-	7.9 ^c^ (0.1)	8.4 ^a^ (0.1)	8.4 ^a^ (0.1)	7.6 ^d^ (0.1)	8.0 ^b^ (0.1)
**Digalactosyl-inositol**	-	-	-	-	-	-	-	-	5.9 ^c^ (0.2)	7.0 ^a^ (0.1)	7.0 ^a^ (0.1)	5.9 ^c^ (0.1)	6.9 ^b^ (0.1)
**Digalactosyl-inositol**	-	-	-	-	-	-	-	-	-	5.2 ^a,b^ (0.1)	5.2 ^a,b^ (0.1)	5.1 ^b^ (0.1)	5.3 ^a^ (0.1)
**Stachyose**	-	-	-	-	-	-	-	-	48.8 ^e^ (0.6)	74.8 ^b^ (0.9)	84.7 ^a^ (5.7)	49.8 ^d^ (0.4)	70.0 ^c^ (1.6)

^a–d^ Different letters indicate significant differences (*p* < 0.05) for samples of each morphological part of alfalfa. - Non-detected.

**Table 4:** Inositol, α-GOS, and sugar concentrations (mg g^−1^) of MAE seeds (Sd2) and leaves (Lv2) lyophilized extracts stored at 50 °C for different times. Standard deviation in brackets (*n* = 3).

	Lv2		Sd2		
Sampling Time (days)	Inositols	Sugars	Inositols	α-GOS	Sugars
0	36.8 (0.6) ^a^	3.5 (0.07) ^a^	28.3 (0.5) ^a^	83.2 (1.0) ^a^	10.5 (0.2) ^a^
5	35.8 (0.2) ^b^	3.16 (0.02) ^b^	27.9 (0.4) ^a,b^	80.8 (1.3) ^b^	8.2 (0.1) ^b^
12	35.0 (0.1) ^b,c^	3.07 (0.08) ^b,c^	27.4 (0.3) ^b,c^	79.8 (1.0) ^b,c^	8.0 (0.1) ^b^
19	34.8 (0.2) ^c,d^	2.98 (0.02) ^c^	27.3 (0.2) ^c^	79.3 (0.5) ^c^	8.0 (0.2) ^b^
26	34.5 (0.1) ^d^	2.81 (0.01) ^c^	27.2 (0.1) ^c^	79.1(0.5) ^c^	7.9 (0.1) ^b^

^a–d^ Different letters indicate significant differences (*p* < 0.05) for each compound at the different storage times. α-GOS, α-galactooligosaccharides.

## Supplementary Materials

The following are available online at https://www.mdpi.com/2304-8158/10/2/346/s1, Table S1: Origin and identification of samples; Table S2: Cyclitols, α-GOS and other sugars identified in alfalfa leaves, stems and seeds extracts obtained by SLE at 75 °C for 16 min using 0.3 g of sample and 10 mL of Milli-Q water. Figure S1: Response surface plots for SLE of A. inositols from Lv2; B. inositols from Sd2; C. α-GOS from Sd2, and for MAE of D. inositols from Lv2, E. inositols from Sd2, and F. α-GOS from Sd2.
