# Supplementary material for: Microwave Assisted Extraction of Bioactive Carbohydrates from Different Morphological Parts of Alfalfa (Medicago sativa L.)
Source: Foods. 2021 Feb 6;10(2):346. doi: 10.3390/foods10020346 (PMC7915009; doi:10.3390/foods10020346)
Supplement: Supplementary file 1 [file foods-10-00346-s001.pdf]

foods MDPI

Supplementary Material

Table S1. Origin and identification of samples.

Sample	Identification	Origin
Leaves	Lv1	San Fernando-Nariño (Colombia)
	Lv2	Ipiales-Nariño (Colombia)
	Lv3	Jongovito-Nariño (Colombia)
	Lv4	Imues-Nariño (Colombia)
Stems	St1	San Fernando-Nariño (Colombia)
	St2	Ipiales-Nariño (Colombia)
	St3	Jongovito-Nariño (Colombia)
	St4	Imues-Nariño (Colombia)
Seeds	Sd1	Semillas Sáenz S.A.S (Colombia)
	Sd2	Semillas RBC S.A.S (Colombia)
	Sd3	El Molino Verde S.A.S (España)
	Sd4	Impulsemillas S.A.S (Colombia)
	Sd5	Bionsan S.C.C.L (España)

Table S2. Cyclitols, α-GOS and other sugars identified in alfalfa leaves, stems and seeds extracts obtained by SLE at 75 °C for 16 min using 0.3 g of sample and 10 mL of Milli-Q water.

Carbohydrate	t_R (min)	I^T
D-Pinitol	5.6	1760
Fructose 1	8.2	1844
Fructose 2	8.6	1856
Ononitol	10.5	1913
Glucose *E*	10.7	1921
Glucose *Z*	11.3	1946
Galactose *E*	10.5	1900
Galactose *Z*	11.6	1945
myo-Inositol	13.8	2048
Sucrose	19.1	2499
Trehalose	19.9	2601
Galactopinitol A	20.1	2610
Galactinol	21.9	2846
Raffinose	24.1	3156
Digalactosyl-inositol	26.0	3465
Digalactosyl-inositol	26.8	3600
Digalactosyl-inositol	27.2	3676
Stachyose	28.7	3950

t_R: retention time. I^T : linear retention index

SLE

Response Surface for Leaves
Sample amount= 0.3 g

A.

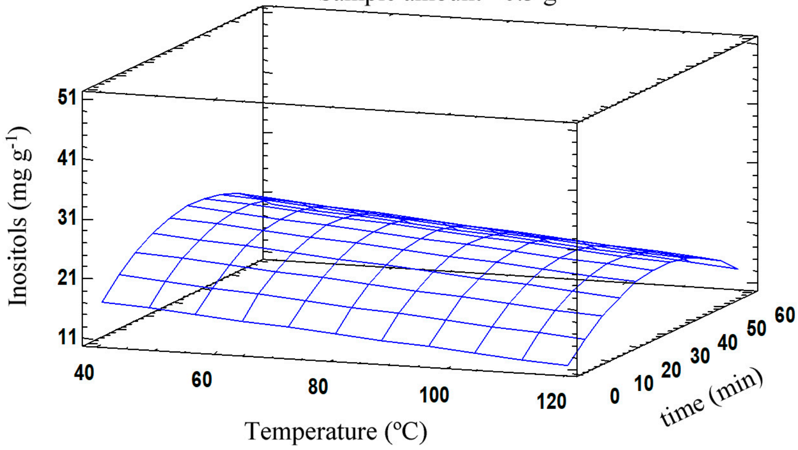

$$y= 4.17428 + 0.0065625*T + 0.566198*t+ 52.0*s – 0.459375*T*s – 0.0871074*t^2$$

Response surface for Seeds
Temperature= 80ºC

B.

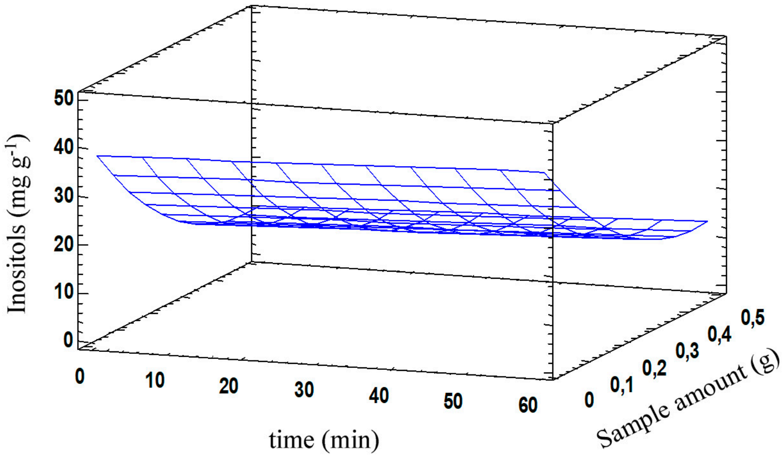

$$y = 38.0054 + 0.0490909*t – 120.411*s + 131.518*s^2$$

Response surface for Seeds
Temperature= 80ºC

C.

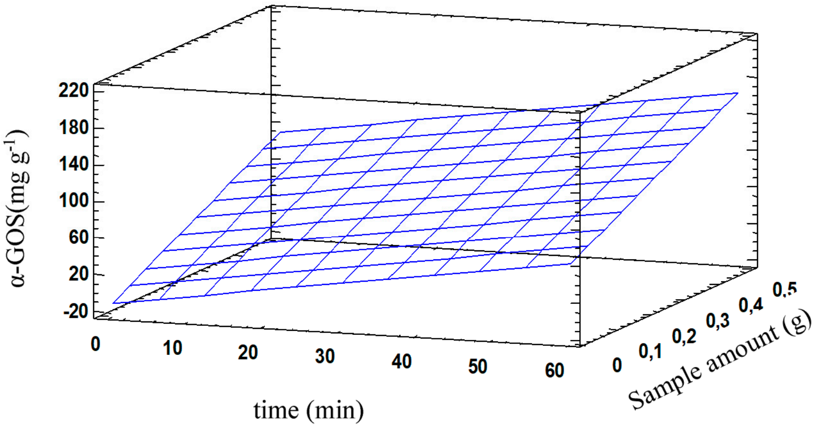

$$y = 12.385 + 1.21*t + 209.0*s$$

D.

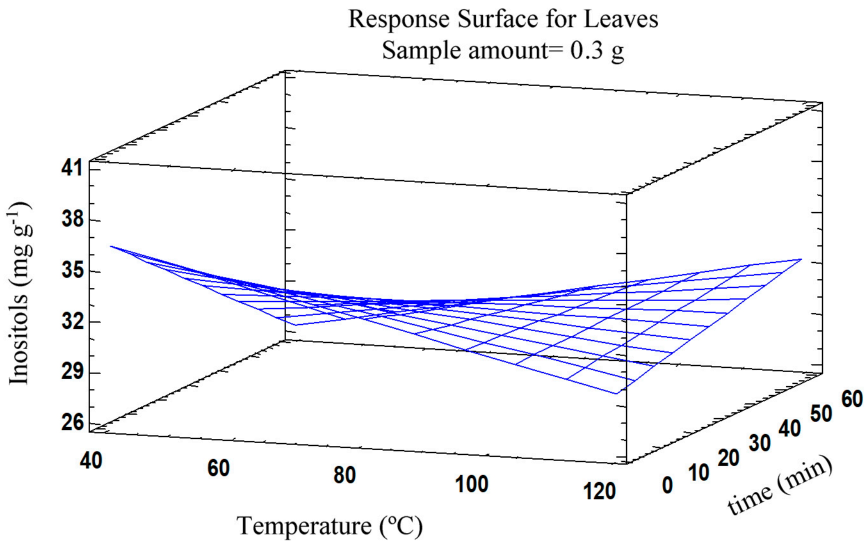

y = 43.6144 – 0.0856818*T – 0.269545*t – 12.625*s + 0.00263636*T*t

E.

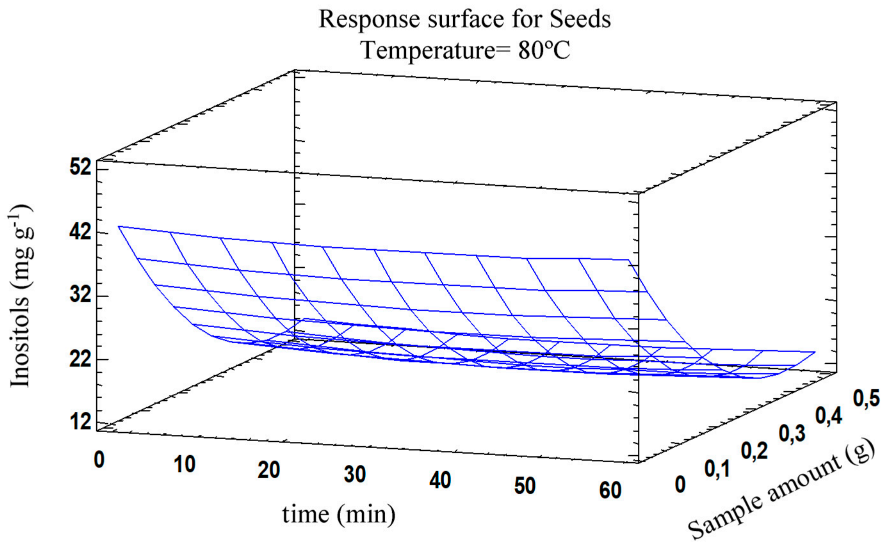

$y = 42.7414 - 0{,}0819835*t - 13.683*s + 0.00126128*t^2 + 160.721*s^2$

F.

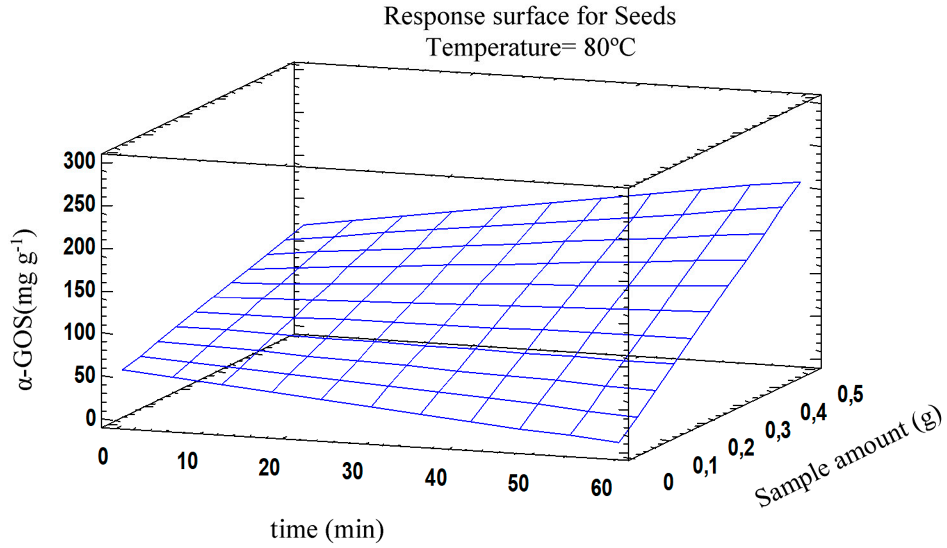

y = 54.9037 – 0.785*t + 13.017*s + 4.50909*t*s

Figure S1. Response surface plots for SLE of **A.** inositols from Lv2; **B.** inositols from Sd2; **C.** α-GOS from Sd2, and for MAE of **D.** inositols from Lv2, **E.** inositols from Sd2, and **F.** α-GOS from Sd2. SLE, solid-liquid extraction; MAE, Mmcrowave Assisted Extraction; α-GOS, α-galactooligosaccharides
